# Gold Nanorod-Incorporated Halloysite Nanotubes Functionalized with Antibody for Superior Antibacterial Photothermal Treatment

**DOI:** 10.3390/pharmaceutics14102094

**Published:** 2022-09-30

**Authors:** Ofer Prinz Setter, Iser Snoyman, Ghazal Shalash, Ester Segal

**Affiliations:** 1 Department of Biotechnology and Food Engineering, Technion—Israel Institute of Technology, Technion City, Haifa 3200003, Israel; 2 The Russel Berrie Nanotechnology Institute, Technion—Israel Institute of Technology, Technion City, Haifa 3200003, Israel

**Keywords:** halloysite, nanoparticles, nanorods, gold, photothermal, bacteria, hybrid, targeting, *E. coli*, antibody

## Abstract

The global spread of antibiotic-resistant strains, and the need to protect the microflora from non-specific antibiotics require more effective and selective alternatives. In this work, we demonstrate for the first time a superior antibacterial photothermal effect of plasmonic gold nanorods (AuNRs) via their incorporation onto natural clay halloysite nanotubes (HNTs), which were functionalized with anti-*E. coli* antibodies (Ab-HNTs). AuNRs were incorporated onto the Ab-HNTs through a facile freeze–thaw cycle, and antibody integrity following the incorporation was confirmed via infrared spectroscopy and fluorescence immunolabeling. The incorporation efficiency was studied using UV-Vis absorption and transmission electron microscopy (TEM). Mixtures of *E. coli* and AuNR-Ab-HNTs hybrids or free AuNRs were irradiated with an 808 nm laser at 3–4 W cm^−2^, and the resulting photothermal antibacterial activity was measured via plate count. The irradiated AuNR-Ab-HNTs hybrids exerted an 8-fold higher antibacterial effect compared to free AuNR under 3.5 W cm^−2^; whereas the latter induced a 6 °C-higher temperature elevation. No significant antibacterial activity was observed for the AuNR-Ab-HNTs hybrid against non-target bacteria species (*Serratia marcescens* and *Staphylococcus epidermidis*). These findings are ascribed to the localization of the photothermal ablation due to the binding of the antibody-functionalized clay to its target bacteria, as supported through TEM imaging. In the future, the HNTs-based selective carriers presented herein could be tailored with other antibacterial nanoparticles or against another microorganism via the facile adjustment of the immobilized antibody.

## 1. Introduction

The overuse of antibiotics poses two main global challenges: the alarming surge in antibiotic resistance [1] and the indiscriminating antibiotic effect also eradicating beneficial indigenous bacterial populations, eventually leading to dysbiosis [2]. Immense research efforts have been directed at developing new antimicrobial solutions to mitigate these challenges [3], including various nanomaterials [4,5,6]. Among these nanomaterials, gold nanoparticles (AuNPs) have shown promising capabilities for externally triggered antibacterial photothermal treatment (APTT), owing to the localized surface plasmon resonance (LSPR) phenomenon [7]. Specifically, gold nanorods (AuNRs) exhibit advantageous high photothermal conversion efficiency at the therapeutic window of the near-infrared (NIR) region, which is not absorbed by most living tissues [8,9]. The mechanism of APTT is still unraveling, with reports indicating thermal damage [10], as well as bacterial membrane disruption (but not necessarily rupture) [11], and even the localized formation of reactive oxygen species [12]. Furthermore, the bulk temperature elevation associated with APTT is ~20 °C lower than that required for simple thermal bacterial inactivation [11,13,14,15]. The lower temperature required is advantageous, as excessive tissue heating and damage could be minimized [11].

Nonetheless, the safety limitations of AuNRs have not yet been sufficiently established [16], and recent studies show that free AuNPs administered locally may eventually spread through the bloodstream and accumulate mostly in the spleen and liver [17]. In addition, colloidal AuNPs tend to aggregate over time, which can in turn alter their intrinsic surface plasmon absorption [18]. To mitigate these shortcomings, it has been proposed to immobilize AuNPs onto a larger particulate system—natural clay halloysite nanotubes (HNTs) [19,20].

HNTs are particles of a mined natural mineral that is low-cost and abundant. These intrinsically mesoporous tubes are composed of geologically rolled alternating layers of alumina and silica exhibiting characteristic dimensions of 600–1000 nm in length, with 50 and 15 nm outer and inner diameters, respectively [21]. The accumulated data regarding HNTs’ biocompatibility suggest similar toxicity thresholds to other high aspect ratio nanomaterials [22,23], and a similar clay mineral, kaolinite, from the same group as HNTs is already in commercial use as an excipient and an active ingredient in dermal formulations [24]. Consequently, HNTs have emerged as a prominent nanomaterial for various biomedical applications, including tableting [25], drug delivery [22,26,27,28,29], tissue engineering [27], bioimaging [30,31], anti-cancer therapy [32,33], and antimicrobial solutions [34]. The latter three have been often realized by utilizing HNTs as ‘mothership’ carriers for cargoes ranging from quantum dots [35,36] through metal oxides NPs to precious metals [19,34], including gold [37,38,39,40,41].

AuNPs can be directly grown on the HNTs surface [20,42] or within their hollow lumen [37,39]. Alternatively, the particles can be loaded onto the clay nanotubes via physical adsorption, avoiding the use of organic solvents or high temperatures [43]. The resulting AuNP–HNT hybrids have shown APTT capabilities against *E. coli* and *Staphylococcus aureus* [37], as well as the protist *Paramecium caudatum* [38], while mild bulk temperatures (<40 °C) have been maintained. In addition, ‘hot spots’ of enhanced LSPR electric fields could be induced through AuNR assemblies [44] incorporated onto the HNTs’ surface [31,38]. Yet, effective APTT requires the selective binding of the plasmonic particles to their target [7,20].

In this work, we combine for the first time the advantages of AuNR immobilization onto HNTs and the modification of HNTs with antibodies against *Escherichia coli* (*E. coli*, as a model microorganism) for targeted APTT. Anti-*E. coli* antibodies were immobilized onto HNTs at their proper orientation using surface-conjugated protein A, as we recently reported [45]. Then, AuNRs were incorporated onto the Ab-functionalized HNTs (Ab-HNTs) using the freezing-induced loading technique, by which the directional growth of ice crystals presses the AuNRs onto the HNTs surface to generate intermolecular adsorptive interactions [43,46]. The resulting AuNR-Ab-HNTs hybrids were thoroughly characterized using Fourier-transform infrared (FTIR) spectroscopy, fluorescence immunolabeling, UV-vis absorption measurements, and transmission electron microscopy (TEM) imaging, including energy dispersive X-ray spectroscopy (EDX). We show that the antibody was successfully immobilized onto the HNTs, and it preserved its antigenic integrity under mild loading conditions. A cytotoxicity assay using a human colon epithelial co-culture (Caco2/HT29) indicated ~90% survival after 24 h exposure to the functionalized clay. The irradiation of the hybrids using a NIR laser (808 nm) resulted in superior antibacterial activity in comparison to free AuNRs, even though the latter induced a 5–2.5-fold higher bulk temperature elevation. Moreover, the irradiated Ab-AuNR-HNTs hybrids did not exert a significant antibacterial effect against non-target bacteria species (*Serratia marcescens* or *Staphylococcus epidermidis*).

We believe that this proof-of-concept demonstration of harnessing antibody-functionalized HNTs as a ‘mothership’ carrier opens the door toward the targeted delivery of a combination of antibacterial nanoparticles (silver or metal oxides) or antibiotics for the localized neutralization of pathogen microorganisms.

## 2. Materials and Methods

### 2.1. Chemicals and Materials

HNTs were provided by NaturalNano (mined at the Atlas Mining Dragon Mine in Salt Lake City, UT, USA), and were dried for 3 h at 150 °C before use. Concentrated sulfuric acid, (3-aminopropyl)triethoxysilane (APTES), succinic anhydride, 1-Ethyl-3-(3-dimethylaminopropyl)carbodiimide (EDC), N-hydroxysulfosuccinimide sodium salt (sulfo-NHS), and ethanolamine were purchased from Sigma-Aldrich Chemicals (St. Louis, MI, USA). The solvents toluene and dimethylformamide (DMF) were purchased from BioLab (Israel), and ethanol absolute was obtained from Gadot Group (Netanya, Israel). Phosphate-buffered saline (PBS) at pH 7.2 0.1 M was prepared by mixing 50 mM disodium hydrogen phosphate (Spectrum Chemicals, New Brunswick, NJ, USA), 17 mM sodium dihydrogen phosphate monohydrate (Merck, Darmstadt, Germany), and 68 mM sodium chloride (BioLab, Jerusalem, Israel). 2-(N-morpholino)ethanesulfonic acid (MES) buffer at pH 6.0, 50 mM, was prepared from 27 mM MES and 23 mM MES sodium salt; both were obtained from Sigma-Aldrich Chemicals (USA). All buffer solutions included Milli-Q water (18.2 MΩ·cm) and were filtered through a 0.22 µm membrane before use. Protein A (PA) from *Staphylococcus aureus* was supplied by Sigma-Aldrich Chemicals (St. Louis, MI, USA), and anti-*E. coli* antibody from rabbit origin was obtained from Virostat (Westbrook, ME, USA). A suspension of gold nanorods (AuNRs, 30 µg mL^−1^) with a diameter and length of 10 and 40 nm, respectively (λmax, 808 nm, dispersion in water, citrate capped) was purchased from Sigma Aldrich (St. Louis, MI, USA). Fluorescein isothiocyanate (FITC)-tagged anti-rabbit immunoglobulin G (IgG) was purchased from Jackson ImmunoResearch Laboratories (West Grove, PA, USA), and bovine serum albumin (BSA) was obtained from MP Biomedicals (Santa Ana, CA, USA). *E. coli* (K-12) and *Serratia marcescens* (ATCC 13880) were generously supplied by the lab of Prof. Sima Yaron (Technion—Israel Institute of Technology). *Staphylococcus epidermidis* (ATCC 14990) were generously supplied by the lab of Prof. Yechezkel Kashi (Technion—Israel Institute of Technology). Luria broth (LB) medium contained 10 g L^−1^ tryptone (BD, Franklin Lakes, NJ, USA), 5 g L^−1^ yeast extract (BD, Franklin Lakes, NJ, USA), 5 g L^−1^ sodium chloride (Biolabs, Jerusalem, Israel), and Milli-Q water (18.2 MΩ·cm). LB agar plates were prepared from LB medium, in addition to 18 g L^−1^ agar (BD). A LIVE/DEAD^®^ BacLight™ Bacterial Viability Kit for microscopy was obtained from invitrogen™ by Thermo Fisher Scientific (Waltham, MA, USA). Glutaraldehyde solution 50 wt.% in water was purchased from Sigma-Aldrich Chemicals (Darmstadt, Germany). Human colon adenocarcinoma Caco-2 cells (ATCC CR2 2101) at passages 19–35 and HT29 cells at passages (11–15) were generously provided by the lab of Prof. Esther Meyron Holtz (Technion—Israel Institute of Technology). Dulbecco’s modified Eagle’s medium (DMEM)—high glucose (Cat. No. D5796) was obtained from Sigma-Aldrich (St. Louis, MI, USA). Fetal bovine serum (FBS), European grade, heat inactivated (South American Origin, Cat. No. 04-127-1A), L-glutamine (200 mM, Cat. No. 03-020-1B), and a mixture of penicillin (10,000 units/mL) and streptomycin (10 mg/mL, Cat. No. 03-031-1B) were purchased from Biological Industries (Jerusalem, Israel). Invitrogen™ alamarBlue™ cell viability reagent was obtained from Thermo Fisher Scientific (Waltham, MA, USA).

### 2.2. Functionalization of HNTs with Anti-E. coli Antibody

Anti-*E. coli* were immobilized onto HNTs, as was previously reported by our group [45]. Briefly, HNTs were first etched under acidic conditions in a solution of sulfuric acid (17 vol.%) at 110 °C for 16 h. After washing with water and drying, the etched HNTs were aminosilanized in an APTES solution in toluene (7 vol.%) at 120 °C for 16 h under reflux. The aminosilanized HNTs were thoroughly washed with toluene and absolute ethanol, and then dried under vacuum. The carboxylation of the amino residues was performed by reacting the aminosilanized HNTs with succinic anhydride in DMF (0.1 M) at room temperature (RT) for 16 h, followed by washes of DMF, water, and absolute ethanol. Sulfo-NHS activation of the carboxylated HNTs was performed via an EDC/sulfo-NHS reaction in MES buffer (40 mg mL^−1^ of both reagents) at RT for 30 min, followed by washes with cold MES and blocking with ethanolamine (100 mM). The PA (0.8 mg mL^−1^) conjugation step was performed at 4 °C for 16 h, and the subsequent antibody immobilization involved the incubation of the PA-conjugated HNTs in a solution of anti-*E. coli* antibody (1 mg mL^−1^) for 16 h at 4 °C, with buffer washes after each step.

### 2.3. AuNR-Ab-HNTs Hybrid Preparation

AuNRs were incorporated onto the Ab-HNTs by adapting the freezing-induced loading method reported by Voronin et al. [43]. In a 2 mL centrifuge tube, 100 µL of Ab-HNTs suspension in PBS (10 mg mL^−1^) was ultrasonicated (ACP-120H ultrasonic bath 3 L 100 W/40 Khz, MRC, Beijing, China) for 3 min and combined with 470 µL of AuNR suspension (30 µg mL^−1^). The resulting mixture (24 µg mL^−1^ Au; 1.7 mg mL^−1^ Ab-HNTs) was frozen at −20 °C for 2 h, thawed at RT, and centrifuged at 825× *g* centrifugation force for 3 min. The supernatant was removed and measured for its absorbance at the range of 350–920 nm using a microplate reader (Varioskan Flash, Thermo Fisher Scientific, USA). The gold content in the hybrids was calculated according to the following Equation (1):

(1) $$\%\;gold\;content=\frac{C_{AuNR\;before\;loading}\cdot V_{AuNR}-C_{AuNR\;after\;loading}\cdot V_{final}}{C_{Ab-HNTs}\cdot V_{Ab-HNTs}}\cdot 100$$

where $C_{AuNR\;before\;loading}$ is the concentration of the AuNR stock suspension according to the supplier (~30 µg mL^−1^), $V_{AuNR}$ is the volume of AuNR suspension added to the loading mixture, $C_{AuNR\;after\;loading}$ is the concentration of the AuNR in the supernatant above the AuNR-Ab-HNTs hybrids, $V_{final}$ is the volume of the final loading mixture, $C_{Ab-HNTs}$ is the concentration of Ab-HNTs in the Ab-HNTs stock suspension, and $V_{Ab-HNTs}$ is the volume of the Ab-HNTs stock suspension added to the loading mixture. AuNR-HNTs (without antibody) were prepared using the same procedure, except with the use of pristine HNTs instead of Ab-HNTs. Hybrid stability was monitored by measuring the absorbance of the suspending medium at 808 nm after storage times of 3 and 48 h at RT, and 4 h at 37 °C under mild shaking (200 RPM).

### 2.4. Infrared Spectroscopy

Each of the HNTs’ modification steps were investigated via attenuated total reflectance Fourier-transform infrared (ATR-FTIR) spectroscopy using a Thermo 6700 FT-IR instrument (USA) with a Smart iTR diamond ATR device at the wavenumber range of 4000–650 cm^−1^. All spectra were normalized to the highest peak at around 1000–1030 cm^−1^, attributed to Si-O bonds of HNTs [47], which are expected to be inert to the chemical modifications performed.

### 2.5. Fluorescence Immunolabeling

To study the antigenic integrity of the immobilized anti-*E. coli* antibody of rabbit origin, the samples were labeled with an anti-rabbit secondary antibody conjugated with fluorescein (FITC). Prior to the labeling, the samples were washed three times with PBS and blocked with BSA (600 µg mL^−1^) for 60 min at RT to minimize non-specific adsorption. The immune-labeling step involved incubation with the FITC-tagged anti-rabbit secondary antibody (15 µg mL^−1^) for 50 min at RT, and three subsequent washes of PBS. Lastly, the samples were imaged under a fluorescence microscope (ZEISS Axio Scope A1, Germany) equipped with a ZEISS (Jena, Germany) Axiocam MRc camera. A constant exposure time of 300 ms was used for all measurements.

### 2.6. Transmission Electron Microscopy and Energy Dispersive X-ray Spectroscopy (EDX)

Samples were mounted on glow discharge-treated carbon type-B grids, and imaged using an FEI Tecnai G2 T20 S-Twin transmission electron microscope (TEM) at an accelerating voltage of 200 kV. Bacteria-containing samples were dehydrated before imaging, via incubation in glutaraldehyde solution (2% in PBS) for 16 h at 4 °C and subsequent treatment with a series of ethanol in water solutions of 10, 25, 50, 75, and 100 vol.%. Elemental mapping was performed using a Titan Themis (Thermo Fisher/FEI) scanning transmission electron microscope (STEM) operated at 200 kV and equipped with a high-angle annular dark-field (HAADF) and Bruker Dual-X EDX detectors.

### 2.7. Cytotoxicity

Cytotoxicity was investigated using the alamarBlue^®^ cell viability assay using a co-culture of human colon adenocarcinoma Caco-2 cells (ATCC CR2 2101, passages 19–35) and HT29 (passages 11–15) at a ratio of 9:1 (Caco-2:HT29). Both cell lines were maintained in Dulbecco’s modified Eagle’s medium (DMEM)—high glucose supplemented with 10% fetal bovine serum (FBS), 2 mM of L-glutamine, 100 unit mL^−1^ penicillin, and 0.01 mg mL^−1^ streptomycin at 37 °C 5% CO_2_. One day prior to exposure, the cells were seeded at 4 × 10^4^ cell cm^−2^ using 200 µL in each well of a 96-well plate. At a confluence of about 80%, the medium was removed and replaced with the following investigated samples: 1.5 mg mL^−1^ AuNR-Ab-HNTs hybrid suspended in medium (expected to contain 20 µg mL^−1^ AuNRs); 1.5 mg mL^−1^ pristine HNTs suspended in medium; and 20 µg mL^−1^ free AuNRs suspended in medium. Wells with untreated cells and no cells served as positive and negative (blank) controls, respectively. Each treatment was studied in three separate wells. At 24 h after exposure, the suspensions above the cells were removed and replaced with 100 µL alamarBlue^®^ solution in medium (0.001 mg mL^−1^). The reaction was carried out for 6.5 h in the dark at 37 °C and 5% CO_2_, and then the colored solutions were transferred into a black 96-well plate for fluorescence measurements using a microplate reader (Varioskan Flash, Thermo Fisher Scientific, USA) at excitation and emission wavelengths of 540 and 585 nm, respectively. A blank reading was deducted from all measurements and the reported viability was relative to the positive control sample (cells grown in growth medium only).

### 2.8. Antibacterial Photothermal Treatment (APTT)

#### 2.8.1. Particle and Bacteria Mixture Preparation

Each bacteria strain was cultured from a single colony in 4 mL of LB overnight at 37 °C, and then set to the log phase using a 1:10 dilution in LB and incubation for 2 h at 37 °C. Bacterial suspensions (7 × 10^7^ cell mL^−1^) were mixed with 1.5 mg mL^−1^ of AuNR-Ab-HNTs hybrid (expected to contain 20 µg mL^−1^ gold), Ab-HNTs, or free AuNRs (20 µg mL^−1^). A 200 µL volume of each particle and bacteria mixture was transferred into a separate well of a polystyrene 96-well plate and then shaken for 2 h to enable adequate interactions between the particles and bacteria.

#### 2.8.2. Near Infrared Irradiation

The studied samples were irradiated with an NIR laser at 808 nm (model WSLS-808-007-H, Wavespectrum Laser Group, Beijing, China) equipped with a fiber collimator (Thorlabs, Newton, NJ, USA). Sample bulk temperature was measured using a thermal camera (FLUKE TiS20 THERMAL IMAGER, Everett, WA, USA) and thermal images were processed using the FLUKE CONNECT software (version 1.1.547.0, Everett, WA, USA). For an example of a full thermal image of the irradiated 96-well plate, please refer to Supporting Information, Figure S1.

### 2.9. Plate Count

Aliquots from the irradiated samples were taken every 3 min (0, 3, 6, 9, and 12 min) and diluted towards a plate count using 8 decimal dilutions in PBS. Each dilution was spread 4 times on an LB agar plate using the drop cast method (3 µL drop). Plates were allowed to dry at RT, and then were incubated overnight at 37 °C. The photothermal activity of free AuNRs was also investigated after 20 min of irradiation at the power intensity range of 2–6 W cm^−2^. For this experiment, 4 decimal dilutions were used and seeded via the drop cast method (10 µL drop). Reduction ratio and log reduction were calculated according to Equations (2) and (3), respectively.

(2) $$Reduciton\;ratio=\frac{PC_{t0}}{PC_{t}}$$

(3) $$Log\;reduciton=\log\left(\frac{PC_{t0}}{PC_{t}}\right)$$

where $PC_{t0}$ is the concentration of bacteria according to plate count before irradiation and $PC_{t}\;$ is the concentration of bacteria according to plate count after *t* min of irradiation.

### 2.10. Live/Dead Cell Staining

The irradiated samples were stained using the LIVE/DEAD^®^ BacLight™ bacterial viability kit at a final concentration of 0.1% for each dye (SYTO^®^ 9 and propidium iodide, Thermo Fisher Scientific) and incubated for 15 min at RT. Fluorescence microscopy images of the samples were taken at a constant exposure time of 500 ms for both the green and red fluorescent channels, in addition to a bright-field image.

### 2.11. Scanning Electron Microscopy

The irradiated samples were fixed with a solution of 2% glutaraldehyde in PBS for 16 h at 4 °C, and gradually dehydrated using a series of ethanol in water solutions (10–100 vol.%). The resulting samples were studied using a Carl Zeiss Ultra Plus (Jena, Germany) high-resolution scanning electron microscope (HR-SEM) at an accelerating voltage of 1.3 keV and a working distance of 3 mm.

## 3. Results and Discussion

### 3.1. Fabrication of AuNR-Ab-HNTs Hybrids

**Scheme 1:**
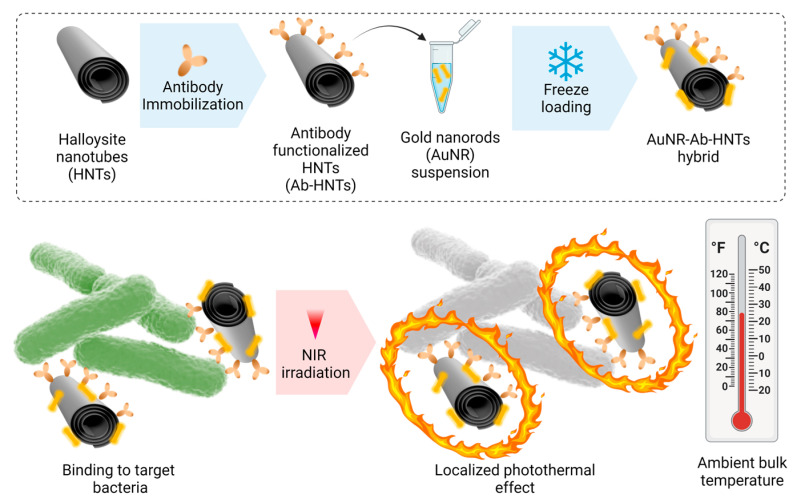
Antibody-functionalized HNTs loaded with AuNRs (AuNR-Ab-HNTs hybrids) for the localized photothermal neutralization of bacteria. The upper panel details the fabrication route, including the immobilization of an antibody onto the HNT surface, followed by the incorporation of the AuNRs onto the functionalized HNTs via the freezing-induced loading technique. The bottom panel describes the concept of HNT targeting of bacteria through antigenic recognition and the consequent localized ablation exerted by the AuNRs upon irradiation, with NIR light neutralizing the bacteria, yet retaining a mild bulk temperature.

HNTs modification consists of two main steps: (1) the surface immobilization of anti-*E. coli* antibody onto a PA-decorated HNTs surface via carbodiimide chemistry [45], and (2) the incorporation of gold nanorods onto HNTs via the freezing-induced loading technique, as schematically illustrated in Scheme 1.

**Figure 1:**
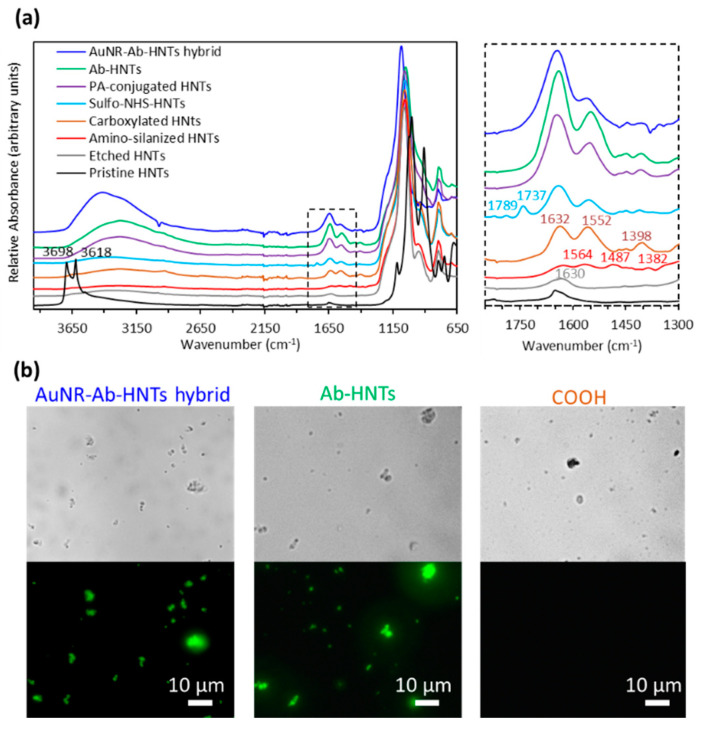
Anti-*E. coli* antibody immobilization onto HNTs and AuNR-Ab-HNTs hybrids. (**a**) FTIR-ATR spectra at the wavenumber range of 4000–650 cm^−1^ (left panel), and a zoom-in view at 1850–1300 cm^−1^ (right panel) for the various synthetic steps: Pristine HNTs (black), Etched HNTs (grey), Amino-silanized HNTs (red), Carboxylated HNTs (brown), Sulfo-NHS-activated HNTs (light blue), PA-conjugated HNTs (purple), Antibody-functionalized HNTs (green), and AuNR-Ab-HNTs hybrids (blue). All spectra are normalized to the inert highest peak in the spectrum. (**b**) Immunolabeling with FITC-anti-rabbit antibody for AuNR-Ab-HNTs hybrids (left panel), antibody-functionalized HNTs before AuNR incorporation (middle panel), and carboxylated HNTs (right panel). Upper panels depict bright-field microscopy images, and the bottom panels show the corresponding green fluorescence micrographs.

The immobilization of anti-*E. coli* antibody onto the HNTs surface was investigated using FTIR-ATR and immunogenic labeling, as depicted in Figure 1a,b, respectively. The FTIR-ATR spectrum of HNTs depicts two characteristic peaks at 3698 and 3618 cm^−1^ ascribed to the Al-OH groups of the alumina portion of HNTs [45,48]. These diminish upon the acidic etching of HNTs, indicating a high degree of alumina removal [29,48]. Aminosilanization of the etched HNTs results in the addition of bands at 1564, 1487, and 1382 cm^−1^ associated with grafted surface amine and CH_2_ deformation [45,47,49]. Following the reaction between the surface amine residues and the succinic anhydride, new bands are observed at 1632, 1552, and 1398 cm^−1^, and are ascribed to the carbonyl residue in a newly formed amide bond and the stretching vibration of a carboxyl residue [45,50]. The spectrum of the reactive Sulfo-NHS intermediate includes two additional bands at 1789 and 1737 cm^−1^, characteristic of cyclic imides [50,51]. The disappearance of these two bands after PA conjugation implies a successful nucleophilic substitution between PA amine and the Sulfo-NHS leaving group [45,50]. Antibody introduction is accompanied by the intensification of the amide bands, suggesting a higher content of protein in the sample, which could be explained by the immobilization of the antibody onto its PA anchor at the proper orientation (see the green trace in Figure 1a [45,52]). No changes in the FTIR spectrum were observed after the AuNRs incorporation (blue trace in Figure 1a); thus, we conclude that this final step did not affect the aforementioned surface modifications, since the metallic AuNRs are not expected to exhibit any FTIR absorption of their own in the range of 3900–650 cm^−1^ [37].

The presence of the immobilized anti-*E. coli* antibody from rabbit origin was also studied through immunolabeling using FITC-anti-rabbit secondary antibody, as displayed in Figure 1b. Green fluorescence was observed for Ab-HNTs, whether before or after the incorporation of AuNRs, indicating that the gentle freezing-induced loading step did not impair the antigenic integrity of the immobilized antibody. The absence of a fluorescent signal from the carboxylated HNTs control supports the specificity of the assay.

**Figure 2:**
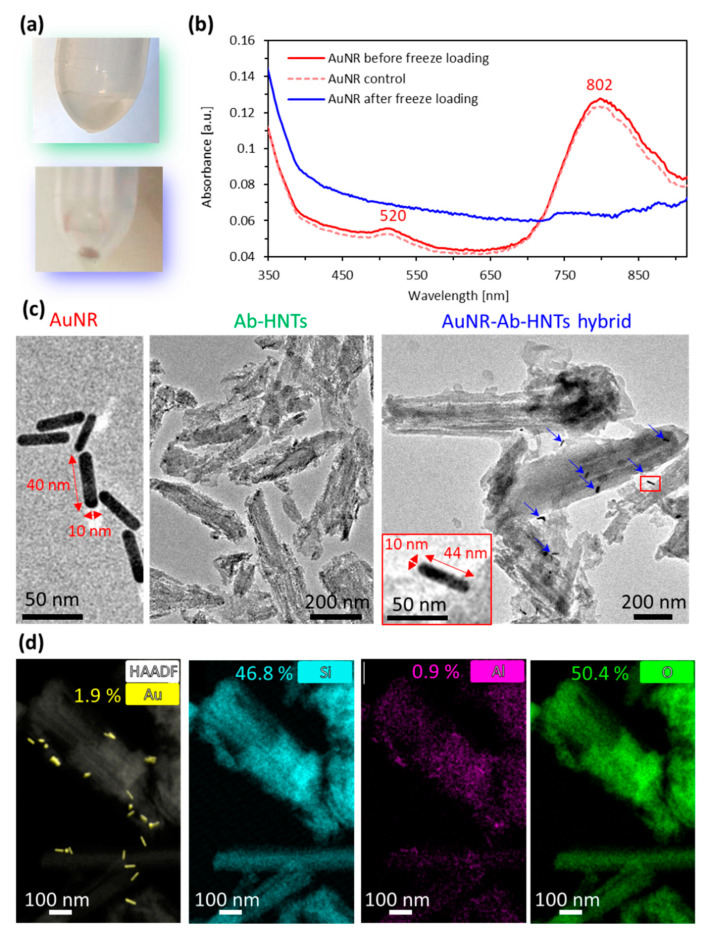
Incorporation of AuNRs onto Ab-HNTs. (**a**) Photographs of the Ab-functionalized HNTs (top panel) and AuNR-Ab-HNTs hybrids (bottom panel); (**b**) UV-Vis spectra at 350–920 nm of AuNR suspension before and after freezing-induced loading (red and blue traces, respectively), and a control of an AuNR suspension with no HNTs similarly processed (light red dashed trace). (**c**) TEM micrographs of: Left panel—free AuNR, red arrows indicate the measured dimensions of a gold nanorod with values detailed next to each corresponding arrow; Middle panel—Ab-HNTs without AuNRs; Right panel—AuNR-Ab-HNTs hybrids, blue arrows mark loaded AuNR, and red-frame inset depicts high magnification of the region of interest marked with a red rectangle. (**d**) STEM-EDX micrographs of AuNR-Ab-HNTs hybrid from left to right: an overlay of high-angle annular dark-field (HAADF) detector and Au elemental mapping; separate elemental mapping for Si, Al, and O. The measured wt.% of each element is indicated next to the element symbol.

Upon freezing-induced loading of AuNRs onto the Ab-HNTs, the thawed treated Ab-HNTs depicted a visual color change from white to purple-blue (see Figure 2a). The incorporation of AuNRs onto the Ab-HNTs was studied by analyzing the UV-Vis absorption of the AuNR suspension before and after the freezing-induced loading process (Figure 2b). The presented spectra show that both the characteristic main absorbance peak at 802 nm, as well as the secondary peak at 520 nm, disappear following the loading process, indicating that the gold nanorods were incorporated onto the HNTs. The respective gold content in the resulting hybrid was calculated according to Equation (1) to be 1.4%. As a control, an AuNR suspension with no HNTs was similarly processed, and only a minor reduction in peak intensity was observed (see Figure 2b). As for the LSPR absorbance characteristics of the AuNR suspension, the peaks at 802 nm and 520 nm well agree with the nanorod length and diameter, respectively (see TEM image in Figure 2c) [53,54].

The morphology of the resulting AuNR-Ab-HNTs hybrids was studied at the nanoscale using TEM (Figure 2c), depicting elongated high-contrast rods on the perimeter of the loaded Ab-HNTs (indicated by the blue arrows in Figure 2c). The dimensions of these rods agree with those of the free AuNRs (see inset in Figure 2c), confirming their successful incorporation onto the HNTs. The Ab-HNTs without AuNRs exhibit a characteristic tubular morphology, with lengths of ~300–600 nm and an outer diameter of ~50 nm [45,48], depicted in Figure 2c (middle panel), where the high contrast rods are not detected. Elemental mapping using STEM-EDX (Figure 2d) further confirms the presence of gold nanorods at the perimeter of the treated Ab-HNTs, which exhibit their characteristic silicon oxide component. Based on this analysis, the gold content in the hybrid was measured to be ~1.9%, in good agreement with the aforementioned composition calculated according to UV-vis absorption (1.4%). Additional comparisons of the elemental compositions of the hybrids and their precursors are included in the Supporting Information (Figure S2).

These findings coincide with previous reports where gold nanoparticles were successfully incorporated onto HNTs using the freezing-induced loading technique. The suggested incorporation mechanism involves the expulsion of the nanoparticles out of the aqueous phase by the crystallization front of the ice, which also forcefully presses the nanoparticles onto the HNTs walls, allowing adsorptive interactions [43,46]. Periodical absorbance measurements at 808 nm for the suspending medium of the hybrids showed no evidence of free AuNR leaching after 48 h of storage at RT and 4 h at 37 °C under mild shaking; see Figure S3 in the Supporting Information.

### 3.2. Cytotoxicity

**Figure 3:**
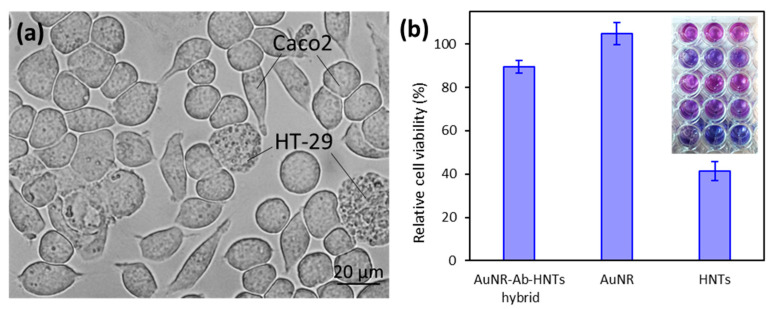
(**a**) Light microscope image of a co-culture of Caco-2 and HT29 cell lines used for cytotoxicity studies. (**b**) Relative viability of the co-culture cells following the exposure of 24 h to AuNR-Ab-HNTs hybrid (1500 µg mL^−1^ functionalized with 20 µg mL^−1^ gold), free AuNR (20 µg mL^−1^), and pristine HNTs (1500 µg mL^−1^), as measured using the alamarBlue^®^ cell viability assay. The viability results are normalized to a co-culture grown in growth media only. Inset showing the relevant section of the 96-well used for the assay with the following samples (top to bottom): cells with only growth medium, pristine HNTs, AuNR, AuNR-Ab-HNTs hybrid, and blank (no cells).

Towards future biomedical applications, the cytotoxicity of the AuNR-Ab-HNTs hybrids was investigated in a co-culture system of human colon adenocarcinoma Caco-2 and mucus-secreting HT29 cells. This co-culture, depicted in Figure 3a, is commonly used at a ratio at of 9:1 (Caco-2: HT29) to mimic the micro-environment of the human colon [55,56,57]. Figure 3b summarizes the cell viability results following exposure to the AuNR-Ab-HNTs hybrids for 24 h, where 89% of the relative viability was measured. The hybrid concentration was kept similar to that used for the photothermal activity studies (1500 µg mL^−1^ HNTs). Notably, exposure to the control pristine HNTs resulted in a 60% reduction in relative cell viability. These results agree with previous reports where the biocompatibility of pristine HNTs has been improved by their coating with polyethylene glycol (PEG) [58], or proteins [39,59]. Moreover, pristine HNTs have been shown to stimulate cell processes related to cell infection and cell injury in a similar Caco-2/HT29 co-culture exposed to a concentration > 10-fold lower (100 µg mL^−1^) than that used in this work [57].

### 3.3. Antibacterial Photothermal Treatment (APTT)

**Figure 4:**
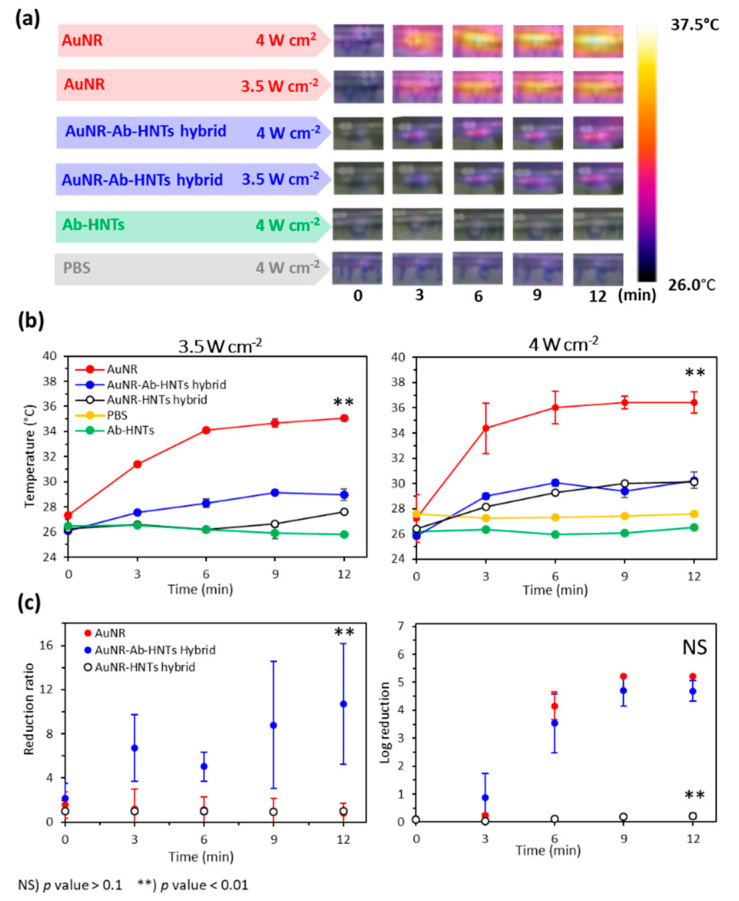
Antibacterial photothermal treatment (APTT) of AuNR-Ab-HNTs hybrids compared with free AuNRs. (**a**) Examples of thermal camera images. (**b**) Temperature measurements using the thermal camera, as a function of irradiation (808 nm) time at a power density of 3.5 and 4 W cm^−2^ (left and right panel, respectively) for AuNR-Ab-HNTs hybrids (blue), free AuNRs (red), Ab-HNTs without AuNRs (green), AuNR-HNTs hybrid without antibody (black with hollow marker), and PBS control (yellow). (**c**) Antibacterial effect measured by plate count as a function of irradiation (808 nm) and time, at a power density of 3.5 and 4 W cm^−2^ for left and right panels, respectively (color scheme similar to all other results in this figure). Error bars represent SD (*n* = 3). NS: *p*-value > 0.1; **: *p*-value < 0.01 according to a two-tailed *t*-test.

The photothermal activity of the resulting AuNR-Ab-HNT hybrids was studied upon irradiation at 808 nm at different power densities. First, the temperature elevations achieved via irradiation were observed using a thermal camera. Thermal images were obtained for AuNR-Ab-HNTs hybrids in suspension, and compared to a suspension of free AuNRs (with equal gold content), Ab-HNTs, and PBS (Figure 4a). The corresponding temperature profiles are presented in Figure 4b. Both the free AuNRs and hybrids exhibited irradiation-induced heating due to LSPR [20,39]. Yet, the attained temperature increase observed for the hybrids (with or without antibody) of up to 2 °C (at 3.5 W cm^−2^) was inferior to the profound heating induced by free AuNRs (of 10 °C, *p* < 0.01). The corresponding calculated photothermal conversion coefficients are 2.0 and 7.8% for the AuNR-Ab-HNTs and free AuNRs, respectively (see calculation in the Supporting Information, page S6). The difference between these coefficients may be ascribed to the effect of the higher thermal resistance in the case of the hybrid, and the shielding effect reported for HNTs [37].

The AuNR-Ab (anti-*E. coli*)-HNT hybrids were mixed in *E. coli* suspensions, and their antibacterial photothermal activity was characterized. The results are summarized in Figure 4c, presenting the bacterial reduction as measured using the plate count method. Interestingly, the irradiated AuNR-Ab-HNTs hybrid exerted a 90% reduction in bacterial viability at a power density of 3.5 W cm^−2^, whereas no bactericidal effect was observed for the suspension of free AuNRs with equal gold content (*p* < 0.01). At a power density of 4 W cm^−2^ (Figure 4c, right panel), both the AuNR-Ab-HNTs hybrids and free AuNRs induced a similar bactericidal effect of ~5 log reduction (*p* > 0.1). This is the first time that such strong photothermal antibacterial activity has been reported for HNTs decorated with gold nanoparticles (see Table S1 in the Supporting Information). Moreover, these values are comparable to those reported for different free AuNR systems in which the achieved temperature increase was 10–30 °C higher than that measured for the hybrids in this study (see Table S2 in the Supporting Information [11,54,60]). The incorporation of gold nanorods onto pristine HNTs, without antibodies, dramatically impaired the latter’s antibacterial photothermal capabilities, probably due to the clustering of AuNRs onto the HNTs’ surface, away from the bacterial cells.

The superior antibacterial activity observed for AuNR-Ab-HNTs hybrids may be ascribed to their antibody-mediated attachment to the target bacteria, leading to the localization of the photothermal ablation, as has been reported for other gold nanoparticles functionalized with capture probes for targeted APTT [7,10,54]. In addition, the LSPR electric fields of AuNR assemblies on HNTs surface could generate electromagnetic ‘hot-spots’ enhancing the plasmonic response, as was recently reported by Kornilova et al. [38]. It should be noted that the temperature values measured in this study (29 °C and 37 °C for hybrids and free AuNRs, respectively) to achieve a 5-log reduction are significantly lower than the established temperature range required for the thermal inactivation of *E. coli* via bulk heating (~54 °C) [14,15]. Thus, the attained bactericidal efficacy in this work may be attributed to the localized ablation and the possible generation of reactive oxygen species (ROS) [7]. Both may consequently induce bacterial membrane rupture or autolysis [7], as well as a bacteriostatic quasi-apoptotic cell death with an intact cell membrane [11].

With irradiation at 3 W cm^−2^ power density (Supporting Information, Figure S4), no significant temperature increment was measured, and no reduction in bacterial viability was observed of samples treated with either AuNR-Ab-HNTs hybrids or free AuNRs. The investigated antibacterial activity could thus be due to the laser irradiation rather than the presence of AuNRs or Ab-HNTs in the sample. For a power density of 2 W cm^−2^, no significant APTT was observed for free AuNRs after 20 min, and for high power densities (5 and 6 W cm^−2^), the measured bulk temperature reached ~40 °C and above (see Supporting Information, Figure S1). Therefore, these power densities were not investigated for the APTTs of AuNR-Ab-HNTs hybrids. No significant heating or bactericidal effects were measured for the controls, including Ab-HNTs (no AuNRs) or only PBS.

**Figure 5:**
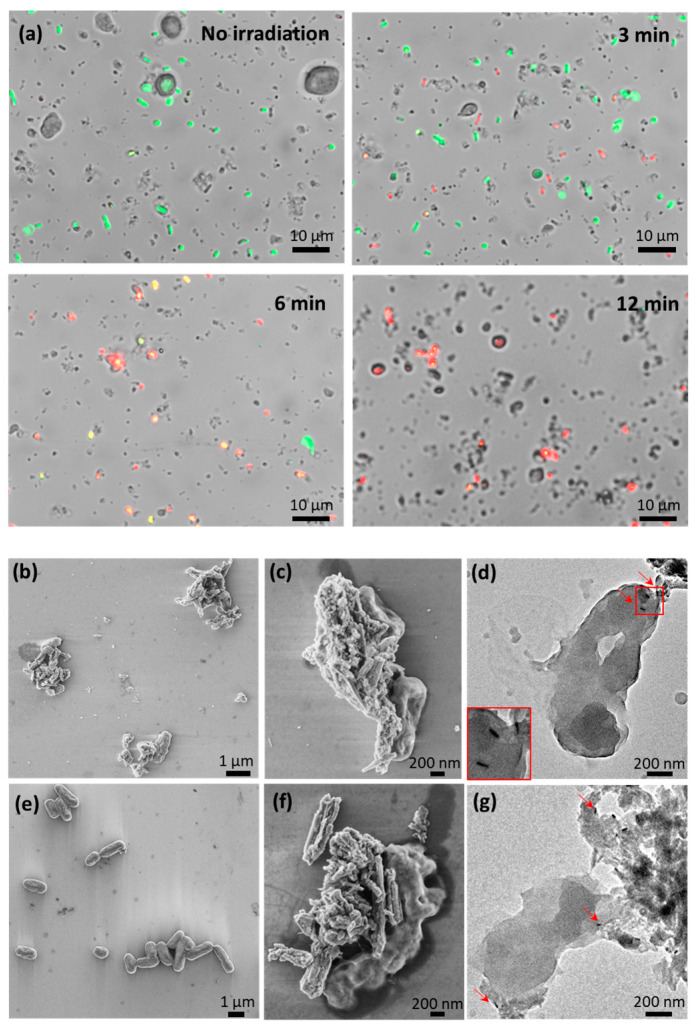
Light and electron microscopy imaging of the antibacterial photothermal effect exerted by AuNR-Ab-HNTs hybrids—mixtures containing AuNR-Ab-HNTs hybrids and *E. coli* irradiated using a 808 nm laser (4 W cm^−2^) and imaged using (**a**) overlay of bright-field and fluorescence microscopy after irradiation time of 0, 3, 6, and 12 min (upper panel left to right and bottom panel left to right, respectively). Bacteria were labeled with Live/Dead staining: Green—Syto9, Red—propidium iodide; (**b**,**c**,**f**) SEM for 12 min irradiation; and (**d**,**g**) TEM for 12 min irradiation. (High contrast gold nanorods are indicated by red arrows. The red frame inset depicts higher magnification of the region of interest marked with a red rectangle.) (**e**) SEM micrograph of untreated *E. coli* cells.

The antibacterial activities of the AuNR-Ab-HNTs hybrids were further investigated at the micro- and nano-scale via light and electron microscopy, respectively. Qualitatively, Live/Dead staining of the hybrid and bacteria mixture (Figure 5a) showed a time-dependent increase in red-fluorescence dead cells upon irradiation, where these cells were observed to be co-aggregated with the Ab-HNTs clusters. This result coincided with the plate count viability measurements discussed earlier (Figure 4c). At a higher magnification, SEM images (Figure 5b,c,f) revealed clusters of HNTs with attached bacteria cells, the latter exhibiting roughened and perforated walls in comparison to the intact untreated cells (Figure 5e). TEM images (Figure 5d,g) provided additional insight as high-contrast gold nanorods (indicated by the red arrows) were observable at the interface between the damaged bacterial cells and HNTs. Similar evidence of such morphological cell damage has been reported for bacterial cells inactivated using plasmonic nano-antimicrobials [7,19].

**Figure 6:**
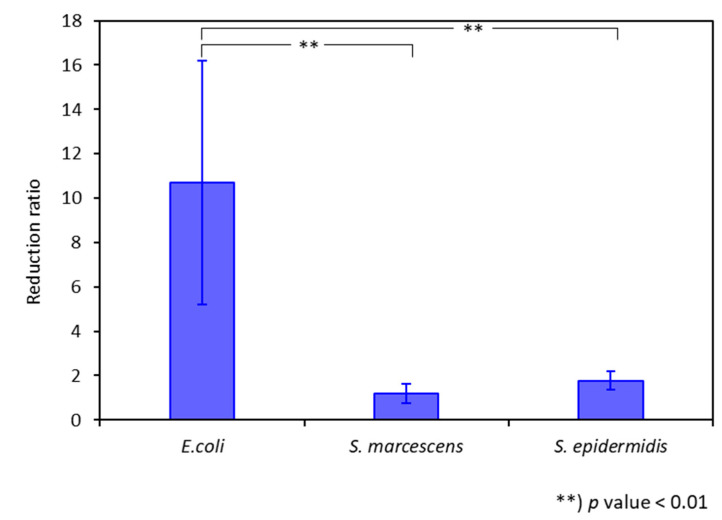
Antibacterial photothermal treatment (APTT) of AuNR-Ab-HNTs hybrids (1.5 mg mL^−1^, expected to contain 20 µg mL^−1^ AuNRs) against three different bacteria strains: *E. coli* (target bacteria), *Serratia marcescens* (*S. marcescens*), and *Staphylococcus epidermidis* (*S. epidermidis*). Irradiation conditions: 808 nm, 3.5 W cm^−2^ after 12 min. **: *p*-value < 0.01 according to a two-tailed *t*-test.

The selectivity of the photothermal activity of the AuNR-Ab-HNTs hybrids was investigated with two non-target bacteria species: *Serratia marcescens* (Gram negative) and *Staphylococcus epidermidis* (Gram positive), and the results are depicted in Figure 6. It is evident that the irradiated AuNR-(anti-*E. coli*) Ab-HNTs hybrids exhibited an order of magnitude higher potency against the target bacteria (*E. coli*), in comparison to the non-target bacterial cells. Therefore, we conclude that the photothermal antibacterial activity of gold nanorods could be selectively enhanced by their incorporation onto antibody-functionalized HNTs.

## 4. Conclusions

This work demonstrates for the first time that antibody-functionalized HNTs incorporated with AuNRs can be tuned to exert superior and selective antibacterial photothermal activity, resulting in up to five orders of magnitude reduction in the target bacteria population. A comparison to free AuNRs shows that upon irradiation, our system induces only mild bulk heating, while its bactericidal efficacy is at least equivalent or improved by almost an order of magnitude (depending on the irradiation power density). In addition, the irradiated hybrids are 90% less harmful to non-target strains (*Serratia marcescens* and *Staphylococcus epidermidis*) in comparison to the target bacteria. This is ascribed to the enhanced localization of the photothermal effect, which is mediated by antibody–antigen recognition between the AuNR-Ab-HNTs hybrids and the target *E. coli* bacteria.

The hybrids are prepared via the facile freezing-induced loading of AuNRs onto antibody-functionalized HNTs. The successful incorporation of the AuNRs onto HNTs was confirmed through UV-Vis absorbance measurements, as well as TEM and EDX studies. ATR-FTIR spectroscopy and fluorescence immunolabeling showed that the immobilized antibody retained its antigenic integrity following the mild freezing-induced loading process. Moreover, the functionalized HNTs were shown to be less cytotoxic than pristine HNTs towards a physiologically relevant co-culture of human colon epithelial cells (Caco-2/HT29).

This proof-of-concept work paves the way for the potential application of the described hybrids for the effective and triggered photothermal neutralization of target bacteria, while the temperature can be kept at physiological conditions. Moreover, these hybrids can potentially serve as ‘mothership’ carriers for any combination of antimicrobial nanoparticles [19,34].

## Data Availability

Not applicable.

## Supplementary Materials

The following supporting information can be downloaded at: https://www.mdpi.com/article/10.3390/pharmaceutics14102094/s1. Figure S1: Antibacterial photothermal treatment (APTT) of free AuNRs; Figure S2: STEM-EDX analysis for AuNR-Ab-HNTs hybrid, Ab-HNTs, and pristine HNTs; Figure S3: Stability monitoring of the AuNR-Ab-HNTs hybrid; Figure S4: Antibacterial photothermal treatment (APTT) of AuNR-Ab-HNTs hybrids compared with free AuNRs at a power density of 3 W cm^−2^, and the calculation of the photothermal conversion efficiency of AuNR (20 µg mL^−1^) and AuNR-Ab-HNTs hybrids; Table S1: Summary of previous reports for HNTs incorporated with gold nanoparticles for photothermal applications; Table S2: Summary of previous reports for gold nanorods utilized for antibacterial applications. References [10,11,13,37,38,39,40,54,60,61,62,63,64,65] are cited in the supplementary materials.
