# Supplementary material for: Gold Nanorod-Incorporated Halloysite Nanotubes Functionalized with Antibody for Superior Antibacterial Photothermal Treatment
Source: Pharmaceutics. 2022 Sep 30;14(10):2094. doi: 10.3390/pharmaceutics14102094 (PMC9611827; doi:10.3390/pharmaceutics14102094)
Supplement: Supplementary file 1 [file pharmaceutics-14-02094-s001.zip › pharmaceutics-1903661-supplementary.pdf]

Supporting Information

Gold Nanorod-Incorporated Halloysite Nanotubes Functionalized with Antibody for Superior Antibacterial Photothermal Treatment

Ofer Prinz Setter [1], Iser Snoyman [1], Ghazal Shalash[1] and Ester Segal [1,2,*]

[1] Department of Biotechnology and Food Engineering, Technion—Israel Institute of Technology, Technion City, Haifa 3200003, Israel

[2] The Russel Berrie Nanotechnology Institute, Technion—Israel Institute of Technology, Technion City, Haifa 3200003, Israel

* Correspondence: esegal@technion.ac.il; Tel: +972-4-829-5071

Table of Contents

Figure S1: Antibacterial photothermal treatment (APTT) of free AuNR	Page S3
Figure S2: STEM-EDX analysis for AuNR-Ab-HNTs hybrid, Ab-HNTs and pristine HNTs	Page S4
Figure S3: Stability monitoring of the AuNR-Ab-HNTs hybrid	Page S5
Calculation of the photothermal conversion efficiency of AuNR (20 µg mL^{-1}) and AuNR-Ab-HNTs hybrid	Page S6
Table S1: Summary of previous reports for HNTs incorporated with gold nanoparticles for photothermal applications	Page S8
Table S2: Summary of previous reports for gold nanorods utilized for antibacterial applications	Page S9
Figure S4: Antibacterial photothermal treatment (APTT) of AuNR-Ab-HNTs hybrids compared with free AuNR at power density of 3 W cm^{-2}	Page S10

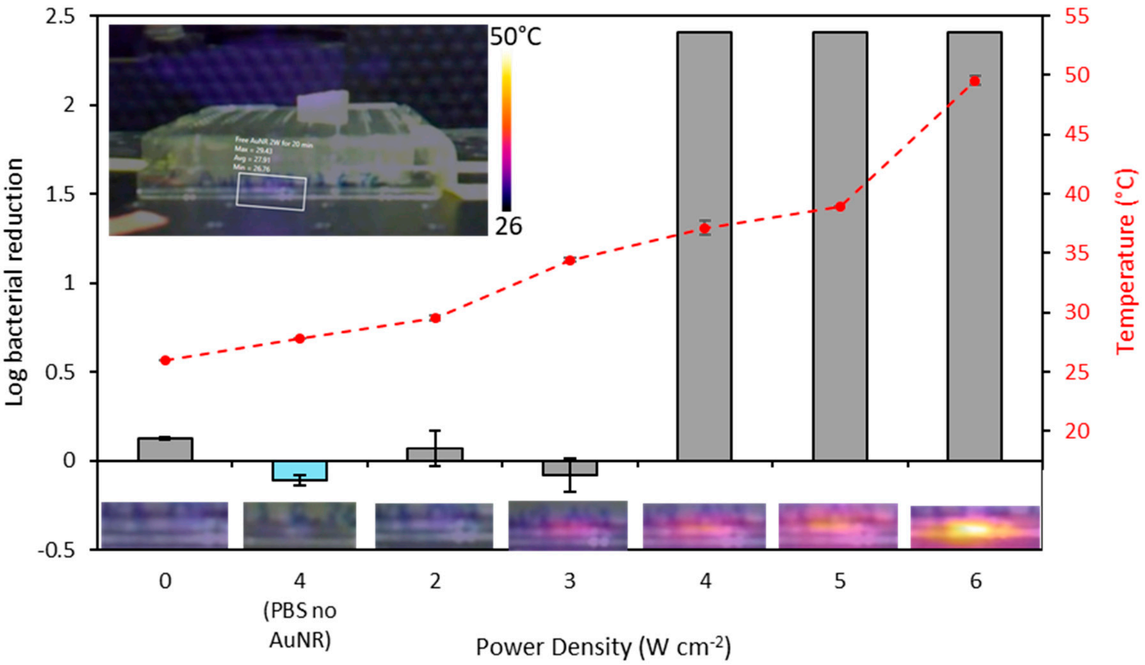

Figure S1. Antibacterial photothermal treatment (APTT) of free AuNR against *E. coli* after 20 min of irradiation (808 nm) at different power density: bulk temperature as measured by a thermal camera (red plot, right axis) and log of bacterial reduction (columns, left axis). Inset depicts the full thermal image of the irradiated well in the 96-well plate.

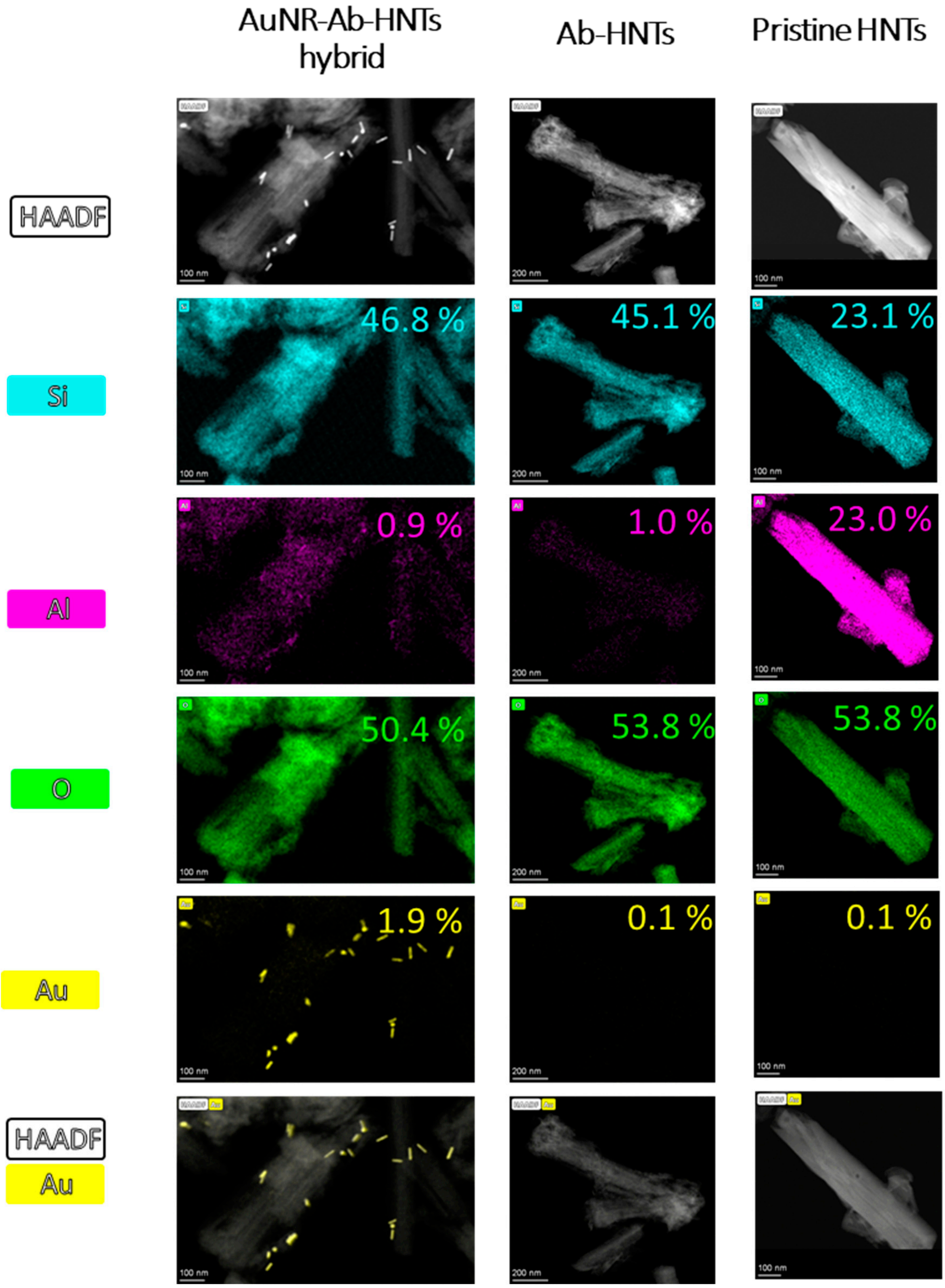

Figure S2. STEM-EDX analysis for AuNR-Ab-HNTs hybrid (left panel), Ab-HNTs (middle panel) and pristine HNTs (right panel) including high-angle annular dark-field (HAADF) image with the corresponding elemental mapping (Si, Al, O, and Au). The measured wt. % of each element is indicated at the right corner of each micrograph.

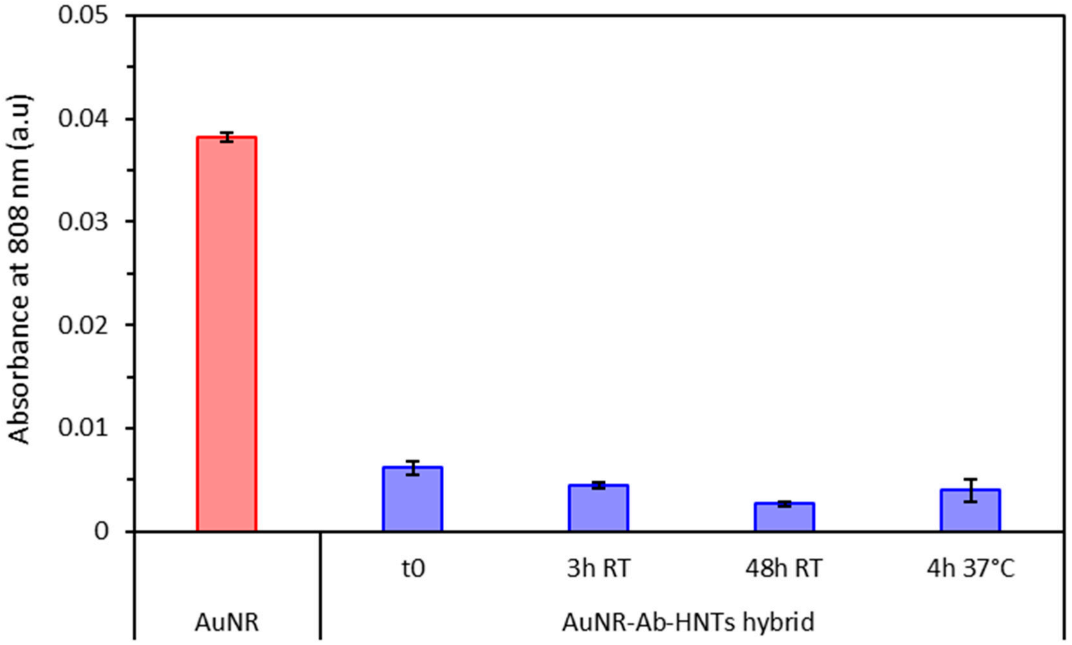

Figure S3. Stability monitoring of the AuNR-Ab-HNTs hybrid by absorbance measurement at 808 nm for the suspending medium at various storage conditions in comparison to free AuNR suspension.

Calculation of the photothermal conversion efficiency of AuNR (20 µg mL^{-1}) and AuNR-Ab-HNTs hybrid (1.5 mg mL^{-1}) under 4 W cm^{-2} irradiation of 808 nm laser [1,2]

During irradiation, heat balance is expressed by Eq. S1:

$$\sum_{i}^{n} m_i Cp_i \frac{dT}{dt} = Q_{NR} + Q_{Dis} - Q_{Surr} \tag{S1}$$

where m_i and Cp_i are the mass and heat capacity of each component in the system, T is the temperature of the whole system, Q_{NR} is the heat generated by the localized surface plasmon resonance (LSPR) effect, Q_{Dis} is the heat dissipated by all the system components other than the gold nanorods that absorb the laser, and Q_{Surr} is the heat conducted to the surroundings by air due to the temperature gradient.

Assuming the mass of the suspended particles is small compared to the PBS in the well, Eq. S1 is simplified into Eq. S2:

$$m_w Cp_w \frac{dT}{dt} = Q_{NR} + Q_{Dis} - Q_{Surr} \tag{S2}$$

where m_w and Cp_w are the mass and heat capacity of water in the irradiated well, given the the values 0.2 g and 4.2 J g^{-1} °C^{-1}, respectively.

When the system reaches maximum temperature, it is at a heat transfer steady state, therefore $\frac{dT}{dt} = 0$ and input energy equals to the heat conducted away by air, described in Eq. S3:

$$Q_{NR} + Q_{Dis} = Q_{Surr} \tag{S3}$$

The laser induced heat input by LSPR, Q_{NR} is given by:

$$Q_{NR} = I(1 - 10^{-A^{808\,nm}})\eta \tag{S4}$$

where I is the laser intensity, $A^{808\,nm}$ is the absorbance of the nanorods at 808 nm, and η

is the photothermal conversion efficiency of the investigated compound.

The heat conduction to the surroundings by air during steady state, $Q_{Surr}$is expressed by the heat transfer equation:

$$Q_{Surr} = hS(T_{max} - T_{surr}) \tag{S5}$$

where h is the heat transfer coefficient, S is the effective area for heat transfer, T_{max} is the maximum temperature of the system during steady state and T_{surr} is the surrounding temperature.

Combining Eq. S3, S4 and S5 gives:

$$\eta = \frac{hS(T_{max} - T_{surr}) - Q_{Dis}}{I(1 - 10^{-A^{808\,nm}})} \tag{S6}$$

Assuming hS is not temperature dependent, it is possible to derive it from the cooling stage when the laser is turned off ($Q_{NR} = 0\ and\ Q_{Dis} = 0$), and Eq. S2 and S5 becomes:

$$
\begin{aligned}
m_w Cp_w \frac{dT}{dt} &= 0 + 0 - hS(T - T_{surr}) \\
\frac{dT}{T - T_{surr}} &= -\frac{hSdt}{m_w Cp_w} \\
\ln\left(\frac{T - T_{surr}}{T_{max} - T_{surr}}\right) &= -\frac{hSt}{m_w Cp_w}
\end{aligned} \tag{S7}
$$

When plotting $-\ln\left(\frac{T-T_{surr}}{T_{max}-T_{surr}}\right)$ against t during the cooling stage we obtain the slope

$$
\begin{aligned}
&\frac{hS}{m_w Cp_w} = 0.0038\ \mathrm{s}^{-1} \\
&hS = 0.0038\ \mathrm{s}^{-1} \cdot 0.2\ g\ \cdot 4.2\ J\ g^{-1}\ °C^{-1} = 0.0032\ W\ °C^{-1}
\end{aligned}
$$

Q_{Dis} is calculated according to the irradiation of only PBS at steady state where $Q_{NR} = 0$, according to Eq. S3 and S5:

$$
0 + Q_{Dis} = hS(T_{max} - T_{surr}) \tag{S8}
$$

In that case T_{max} was 28°C (see Figure 4b in the manuscript) and T_{surr} was 26 °C, and Q_{Dis} thus calculated to be 0.0063 W.

Combining the calculated hS and Q_{Dis}, it is possible to calculate η according to Eq. 6 given that under 4 W cm^{-2}, T_{max} was measured to be 36 and 30 °C for AuNR and Ab-AuNR-HNTs hybrid, respectively (see Figure 4b in the manuscript) and the surrounding temperature was 26 °C. I is 4 W cm^{-2} x 0.32 cm^{2} (well area) = 1.28 W, and $A^{808\ nm}$ is 0.13 (see Figure 2b in the manuscript).

Thus,

$$
\eta_{AuNR} = \frac{hS(T_{max} - T_{surr}) - Q_{Dis}}{I(1 - 10^{-A^{808\ nm}})} = \frac{0.0032\ W\ °C^{-1}(36 - 26°C) - 0.0063\ W}{1.28\ W(1 - 10^{-0.13})} * 100 = \mathbf{7.8}\%
$$

$$
\eta_{AuNR\text{-}Ab\text{-}HNTs} = \frac{hS(T_{max} - T_{surr}) - Q_{Dis}}{I(1 - 10^{-A^{808\ nm}})} == \frac{0.0032\ W\ °C^{-1}(30 - 26°C) - 0.0063\ W}{1.28\ W(1 - 10^{-0.13})} * 100 = \mathbf{2.0}\%
$$

Table S1. Summary of previous reports for HNTs incorporated with gold nanoparticles for photothermal applicaitons

Sample	Gold particles	Application	Concentration	Irradiation	Maximum Temperature (°C)	Antibacterial	Ref
Gold nanoparticles in HNTs-chitin hydrogel	Round 5-10 nm synthesized inside HNTs lumen	Wound healing	Au: unknown<br>HNTs: 12 mg mL^{-1}	808 nm, 0.8-2.2 W cm^{-2} 5-8 min	40	*E. coli, S. aureus*<br>10^6 cell mL^{-1} 2 log reduction	[3]
Gold nanoparticles on HNTs	Round 10 nm synthesized onto HNTs	Antimicrobial	Au: 50 µg mL^{-1}<br>HNTs: 1 mg mL^{-1}	Continuous: 515 nm, 1 W cm^{-2}, 180 min<br>Pulse: 532 nm 20 ns 12 W cm^{-2}	25	*Paramecium caudatum*<br>2 log reduction	[4]
Gold nanorods in HNTs loaded with doxorubicin	Rods synthesized inside HNTs lumen. HNTs modified with folic acid	Antitumor	Au: 20 µg mL^{-1}<br>HNTs: 0.4 mg mL^{-1}	808 nm, 1 W cm^{-2}, 8 min	58	N/A	[5]
Gold nanoparticle or continuous shell on HNTs	Round 20-40 nm particles or a continuous shell synthesized onto HNTs	N/A	Au: 40 µg mL^{-1}<br>HNTs: 2 mg mL^{-1}	808 nm, 0.8 W cm^{-2}, 12 min	32	N/A	[6]

Table S2. Summary of previous reports for gold nanorods utilized for antibacterial applications

Sample	Concentration (µg mL^{-1})	Irradiation	Maximum Temperature (°C)	Bacteria	Antibacterial Effect	Ref
Hydrophilic and hydrophobic<br>AuNR length: 40, diameter: 10 nm	Hydrophilic: 8<br>Hydrophobic: 170	808 nm, 3 W cm^{-2} 15 min	Hydrophilic: 60<br>Hydrophobic: 55 (on agar)	*Staphylococcus aureus*<br>*Propionibacterium acnes*	4 log reduction<br>Initial conc: 10^{6} cell mL^{-1}	[7]
Silica coated AuNR<br>length: 40, diameter: 15 nm	30	808 nm, 3 W cm^{-2} up to 5 min	45-61	*E. coli*	50% - 2 log reduction<br>Initial conc: 10^{6} cell mL^{-1}	[8]
AuNR<br>length: 33, diameter: 7 nm	6<br>2	810 nm, ~0.6 W cm^{-2} 20 min	65	*Staphylococcus aureus*<br>*E. coli*	4 log reduction<br>Initial conc: 10^{4} cell mL^{-1}	[9]
AuNR functionalized with antibodies<br>length: 68, diameter: 18 nm	Unknown	785 nm, 0.08 W (unknown area), 5 min	Unknown	*Pseudo aeruginosa*	80 % reduction	[10]
AuNR functionalized with protease<br>length: 32, diameter: 8 nm	50<br>40	808 nm, 2 W cm^{-2}, 20 min	~60	*Staphylococcus aureus*<br>*E. coli*	2 log reduction<br>Initial conc: 10^{6} cell mL^{-1}	[11]
Phospholipid-decorated gold nanorods<br>length: 50, diameter: 12 nm	7.5	808 nm , 3 W cm^{-2}, 15 min	62	*Pseudomonas aeruginosa*	6 log reduction<br>Initial conc: 10^{6} cell mL^{-1}	[12]
AuNR modified with an Ag and Pt shell<br>Length: 60, diameter: 20	20	808 nm , 22 W cm^{-2}, 5 min	65	*E. coli*	7 log reduction<br>Initial conc: 10^{8} cell mL^{-1}	[13]
AuNR<br>Length: 50, diameter: 13	10	785 nm , 0.05 W cm^{-2}, 10 min	44	*E. coli*	50 % reduction<br>Initial conc: 10^{3} cell mL^{-1}	[14]
AuNR modified with an Ag shell<br>Length: 50, diameter: 13					2 log reduction	

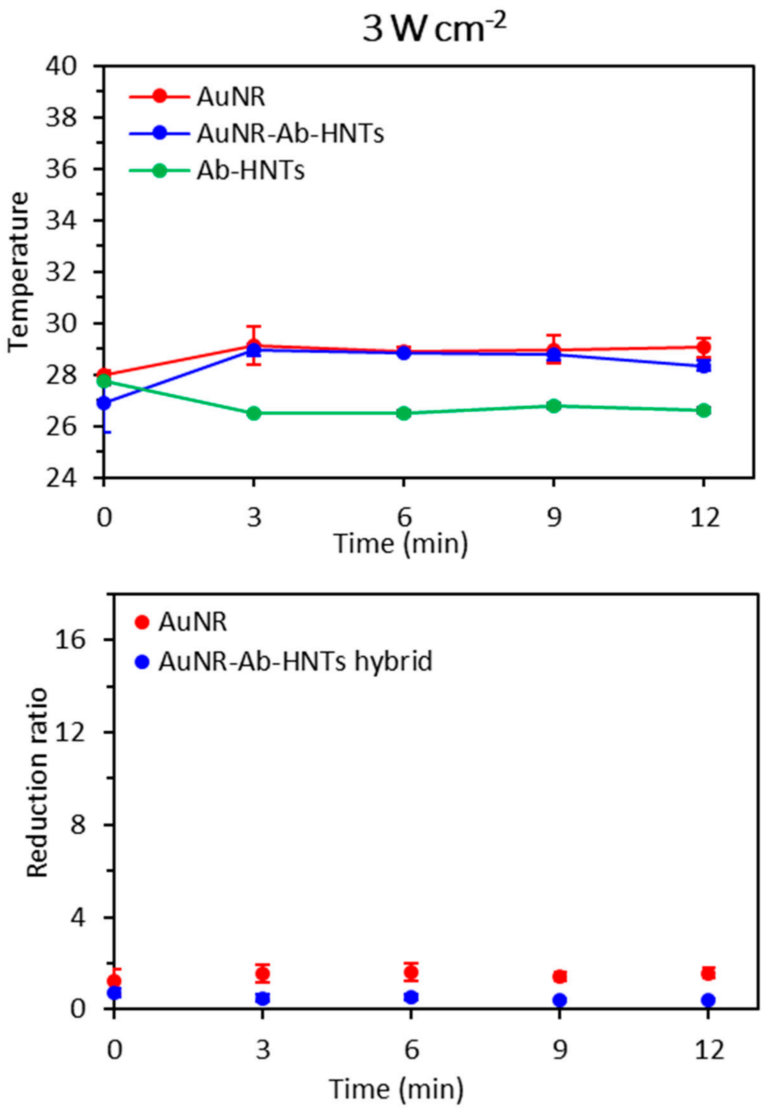

Figure S4. Antibacterial photothermal treatment (APTT) of AuNR-Ab-HNTs hybrids compared with free AuNR at power density of 3 W cm^{-2}. Upper panel – temperature measurements by a thermal camera as a function of irradiation (808 nm) time for AuNR-Ab-HNTs hybrids (blue), free AuNR (red), and Ab-HNTs without AuNR (green). Lower panel – antibacterial effect measured by plate count as a function of irradiation (808 nm) time (color scheme similar to all other results in this figure). Error bars represent SD (n=3).

References:

1. Tian, Q.; Jiang, F.; Zou, R.; Liu, Q.; Chen, Z.; Zhu, M.; Yang, S.; Wang, J.; Wang, J.; Hu, J. Hydrophilic Cu9S5 Nanocrystals: A Photothermal Agent with a 25.7% Heat Conversion Efficiency for Photothermal Ablation of Cancer Cells in Vivo. *ACS Nano* **2011**, *5*, 9761–9771, doi:10.1021/NN203293T.

2. Liu, X.; Li, B.; Fu, F.; Xu, K.; Zou, R.; Wang, Q.; Zhang, B.; Chen, Z.; Hu, J. Facile Synthesis of Biocompatible Cysteine-Coated CuS Nanoparticles with High Photothermal Conversion Efficiency for Cancer Therapy. *Dalton Transactions* **2014**, *43*, 11709–11715, doi:10.1039/C4DT00424H.

3. Zhao, P.; Feng, Y.; Zhou, Y.; Tan, C.; Liu, M. Gold@Halloysite Nanotubes-Chitin Composite Hydrogel with Antibacterial and Hemostatic Activity for Wound Healing. *Bioact Mater* **2023**, *20*, 355–367, doi:10.1016/J.BIOACTMAT.2022.05.035.

4. Kornilova, A. v.; Kuralbayeva, G.A.; Stavitskaya, A. v.; Gorbachevskii, M. v.; Karpukhina, O. v.; Lysenko, I. v.; Pryadun, V. v.; Novikov, A.A.; Vasiliev, A.N.; Timoshenko, V.Y. Gold Nanoparticles Immobilized on Halloysite Nanotubes for Spatially-Temporally Localized Photohyperthermia. *Appl Surf Sci* **2021**, *566*, doi:10.1016/J.APSUSC.2021.150671.

5. Zhang, J.; Luo, X.; Wu, Y.P.; Wu, F.; Li, Y.F.; He, R.R.; Liu, M. Rod in Tube: A Novel Nanoplatform for Highly Effective Chemo-Photothermal Combination Therapy toward Breast Cancer. *ACS Appl Mater Interfaces* **2019**, *11*, 3690–3703, doi:10.1021/ACSAMI.8B17533.

6. Zieba, M.; Hueso, J.L.; Arruebo, M.; Martínez, G.; Santamaría, J. Gold-Coated Halloysite Nanotubes as Tunable Plasmonic Platforms. *New Journal of Chemistry* **2014**, *38*, 2037–2042, doi:10.1039/C3NJ01127E.

7. Mahmoud, N.N.; Alkilany, A.M.; Khalil, E.A.; Al-Bakri, A.G. Nano-Photothermal Ablation Effect of Hydrophilic and Hydrophobic Functionalized Gold Nanorods on Staphylococcus Aureus and Propionibacterium Acnes. *Scientific Reports 2018 8:1* **2018**, *8*, 1–10, doi:10.1038/s41598-018-24837-7.

8. Qiang, L.; Jin, H.; Feng, Y.; Wu, R.; Song, Y.; Liu, L. Apoptosis-like Bacterial Death Modulated by Photoactive Hyperthermia Nanomaterials and Enhanced Wound Disinfection Application. *Nanoscale* **2021**, *13*, 14785–14794, doi:10.1039/D1NR02881B.

9. Castillo-Martínez, J.C.; Martinez-Castanon, G.A.; Martínez-Gutierrez, F.; Zavala-Alonso, N.V.; Patiño-Marín, N.; Niño-Martinez, N.; Zaragoza-Magaña, V.; Cabral-Romero, C. Antibacterial and Antibiofilm Activities of the Photothermal Therapy Using Gold Nanorods against Seven Different Bacterial Strains. *J Nanomater* **2015**, *2015*, doi:10.1155/2015/783671.

10. Norman, R.S.; Stone, J.W.; Gole, A.; Murphy, C.J.; Sabo-Attwood, T.L. Targeted Photothermal Lysis of the Pathogenic Bacteria, Pseudomonas Aeruginosa, with Gold Nanorods. *Nano Lett* **2008**, *8*, 302–306, doi:10.1021/nl0727056.

11. Li, W.; Geng, X.; Liu, D.; Li, Z. Near-Infrared Light-Enhanced Protease-Conjugated Gold Nanorods As A Photothermal Antimicrobial Agent For Elimination Of Exotoxin And Biofilms. *Int J Nanomedicine* **2019**, *14*, 8047–8058, doi:10.2147/IJN.S212750.

12. Al-Bakri, A.G.; Mahmoud, N.N.; Estelrich, J.; Busquets, M.A. Photothermal-Induced Antibacterial Activity of Gold Nanorods Loaded into Polymeric Hydrogel against Pseudomonas Aeruginosa Biofilm. **2019**, doi:10.3390/molecules24142661.

13. Hu, X.; Zhao, Y.; Hu, Z.; Saran, A.; Hou, S.; Wen, T.; Liu, W.; Ji, Y.; Jiang, X.; Wu, X. Gold Nanorods Core/AgPt Alloy Nanodots Shell: A Novel Potent Antibacterial Nanostructure. *Nano Research 2013 6:11* **2013**, *6*, 822–835, doi:10.1007/S12274-013-0360-4.

14. Hu, B.; Wang, N.; Han, L.; Chen, M.L.; Wang, J.H. Core–Shell–Shell Nanorods for Controlled Release of Silver That Can Serve as a Nanoheater for Photothermal Treatment on Bacteria. *Acta Biomater* **2015**, *11*, 511–519, doi:10.1016/J.ACTBIO.2014.09.005.
